# Supplementary material for: Accelerating dynamics of collective attention
Source: Nat Commun. 2019 Apr 15;10:1759. doi: 10.1038/s41467-019-09311-w (PMC6465266; doi:10.1038/s41467-019-09311-w)
Supplement: Supplementary file 1 — Supplementary Information [file 41467_2019_9311_MOESM1_ESM.pdf]

Supplementary information for
Accelerating Dynamics of Collective Attention

Lorenz-Spreen et al.

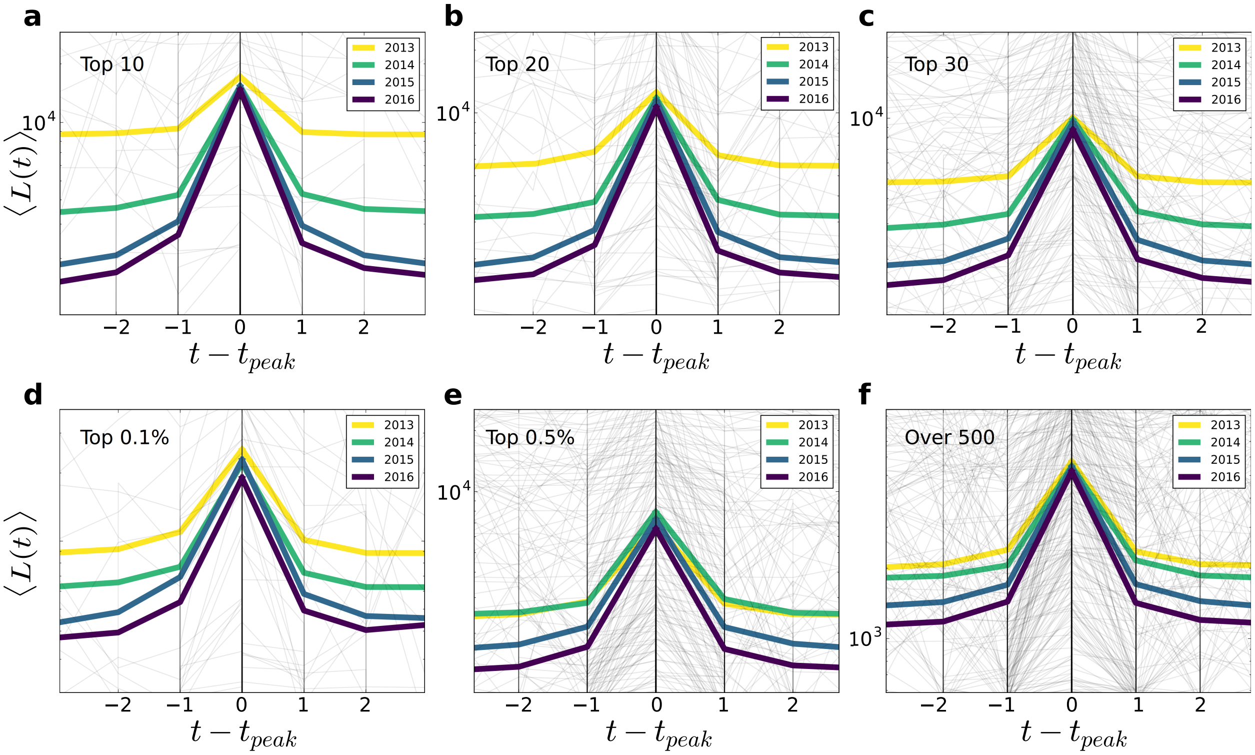

Supplementary Figure 1: **Average trajectories around a maximum for different top groups on Twitter:** (2013-2016) **a** Top 10 hashtags of every hour **b** Top 20 hashtags of every hour **c** Top 30 hashtags of every hour.**d** Relative top group: Top 0.1% hashtags of every hour and **e** top 0.5% of all hashtags. **f** Threshold top group: Hashtags that have been used more than 500 times in every hour.

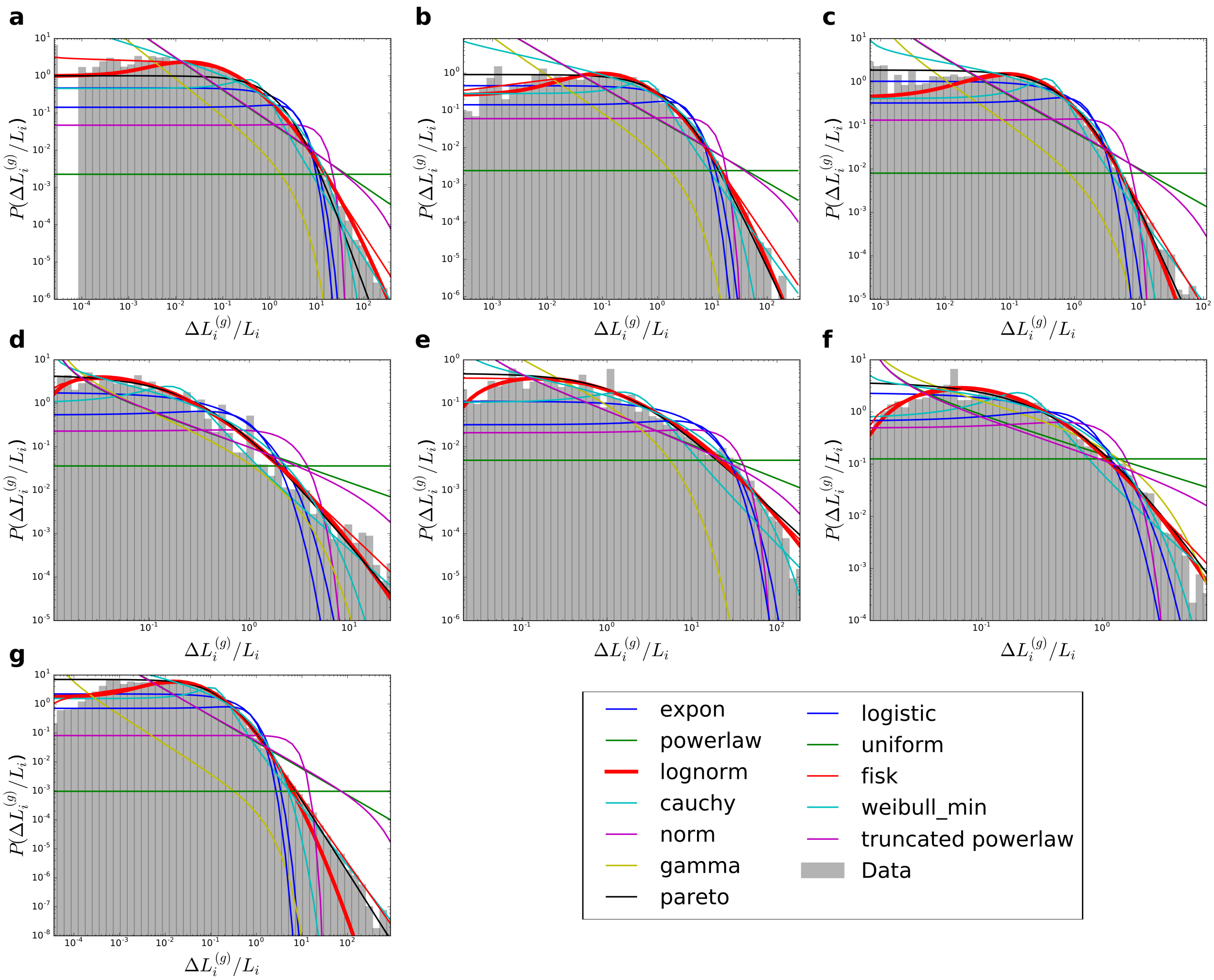

Supplementary Figure 2: **Testing candidate distribution functions:** Comparison of different probability density function, which are fitted to the empirical data, for all the datasets under investigation (**a-g** as given in the legend). The log-normal distribution has the lowest residual sum of squares across the datasets and is marked with the thick red line. Other good candidates are the Fisk, the Weibull and the Pareto distribution.

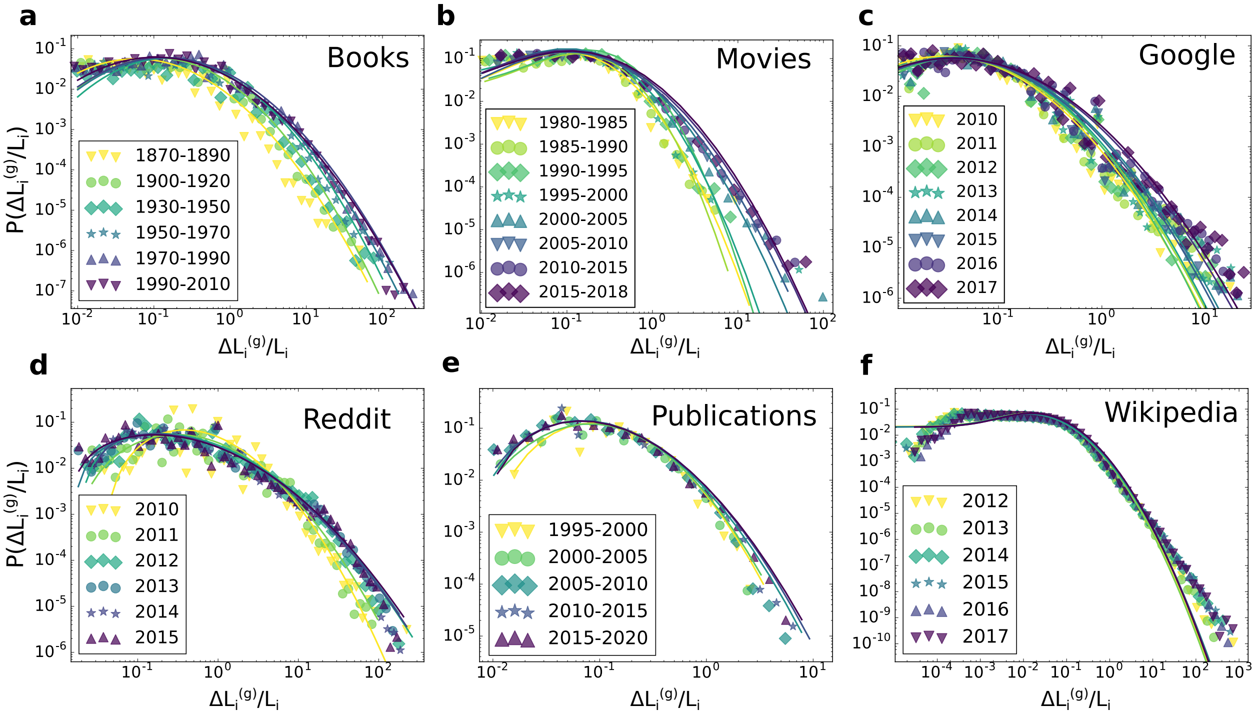

Supplementary Figure 3: **Distributions of gains** $P(\Delta L_{\mathrm{i}}^{(\mathbf{g})}/L_{\mathrm{i}})$**:** The long-term changes in the distribution of relative gains $[\Delta L_{\mathrm{i}}^{(\mathrm{g})}/L_{\mathrm{i}}](t) = (L_{\mathrm{i}}(t) - L_{\mathrm{i}}(t-1))/L_{\mathrm{i}}(t+1) > 0$ for all other datasets. **a** Gain distribution of n-gram counts in Google Books. **b** Gains in box-office sales of movies. **c** Gains in relative search queries on Google Trends. **d** Gains in comment count on Reddit. **e** Gains in citation count in the APS-corpus. **f** Gains of traffic on English Wikipedia articles.

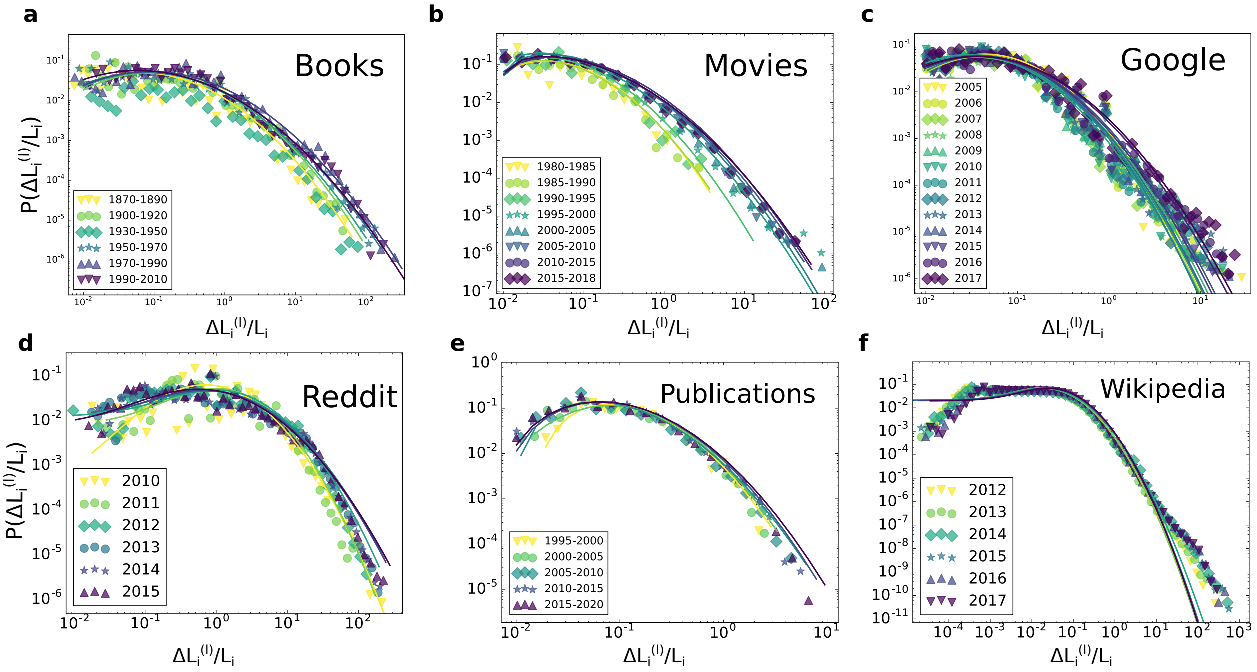

Supplementary Figure 4: **Distributions of losses** $P(\Delta L_{\mathrm{i}}^{(\mathbf{l})}/L_{\mathrm{i}})$**:** The distribution of relative losses $[\Delta L_{\mathrm{i}}^{(\mathrm{l})}/L_{\mathrm{i}}](t) = (L_{\mathrm{i}}(t) - L_{\mathrm{i}}(t+1))/L_{\mathrm{i}}(t+1) > 0$ for the other datasets. **a** Losses distribution of n-gram counts in Google Books. **b** Losses in box-office sales of movies. **c** Losses in relative search queries on Google Trends. **d** Losses in comment count on Reddit. **e** Losses in citation count in the APS corpus. **f** Losses of traffic on English Wikipedia articles.

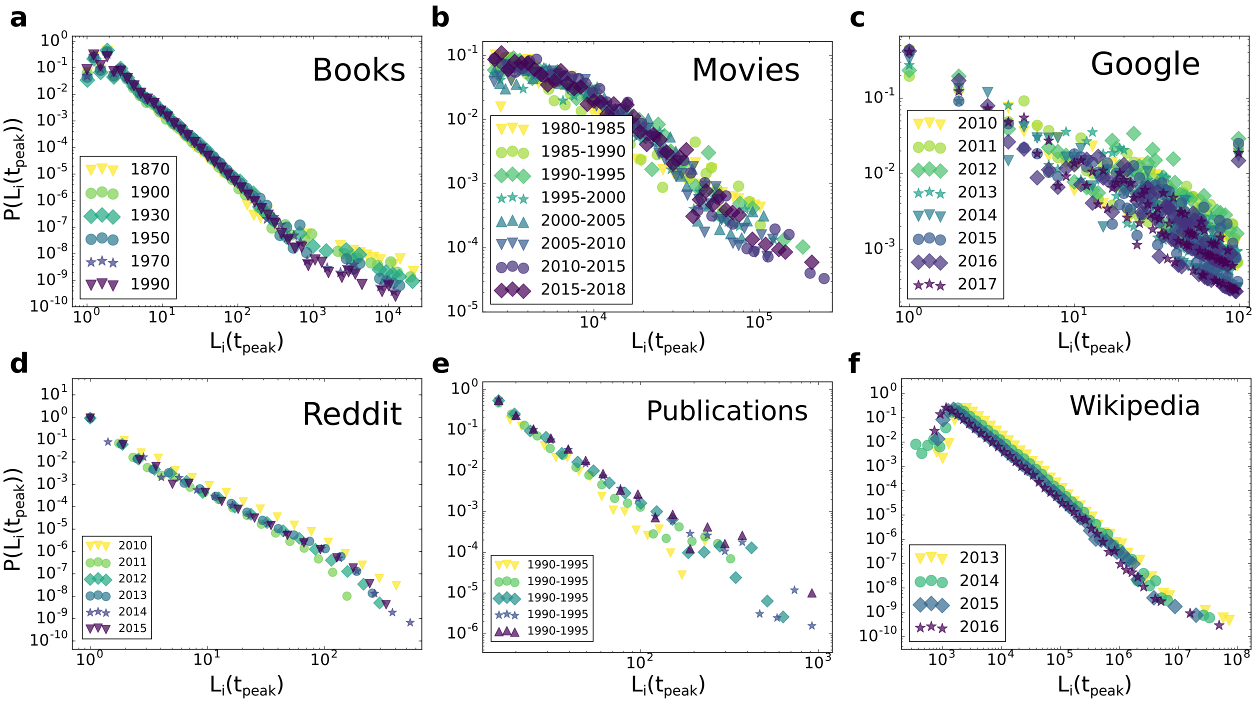

Supplementary Figure 5: **Distributions of maxima** $P(L_\mathrm{i}(t_{\mathbf{peak}}))$**:** **a** Peak height distribution of n-gram counts in Google Books. **b** Peak height distribution of box-office sales. **c** Peak height distribution relative search queries on Google Trends (here the value 100 stands out, because these are the maxima of each category used as a normalization). **d** Peak heights from the Reddit dataset **e** Distribution of maxima from the publication dataset (here the development towards more citations in general can be observed). **f** Distribution for the maximum of visitors wihtin each hour on English Wikipedia articles.

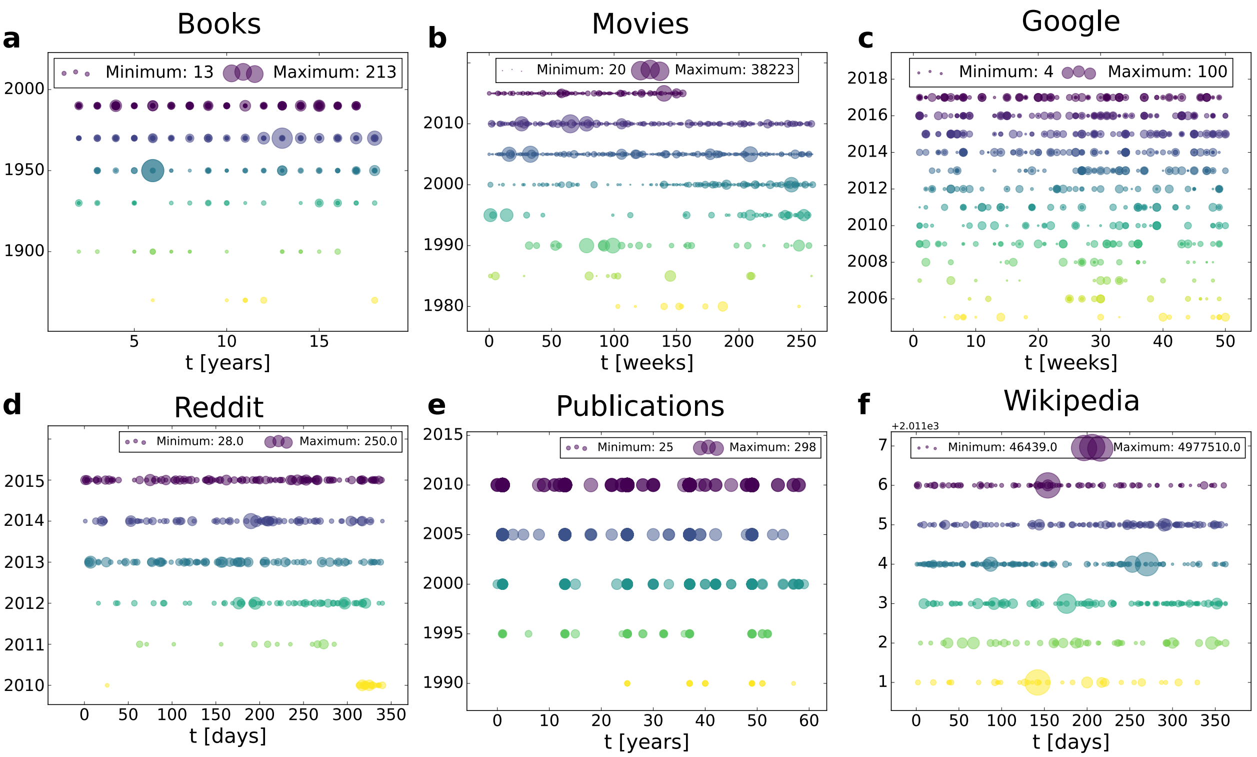

Supplementary Figure 6: **Burst events over time:** The timing of extreme events in different media. A dot is plotted whenever the a relative increase exceeds a threshold $[\Delta L_{\mathrm{i}}^{(\mathrm{g})}/L_{\mathrm{i}}](t_{\mathrm{burst}}) > \delta$ and is followed by a steep decline $[\Delta L_{\mathrm{i}}^{(\mathrm{l})}/L_{\mathrm{i}}](t_{\mathrm{burst}}) > \delta$. **a** Google Books, $\delta = 12.0$, **b** Movies, $\delta = 1.5$, **c** Google Trends, $\delta = 2.0$, **d** Reddit $\delta = 25.0$, **e** Citations $\delta = 1.0$, **f** Wikipedia, $\delta = 35.0$.

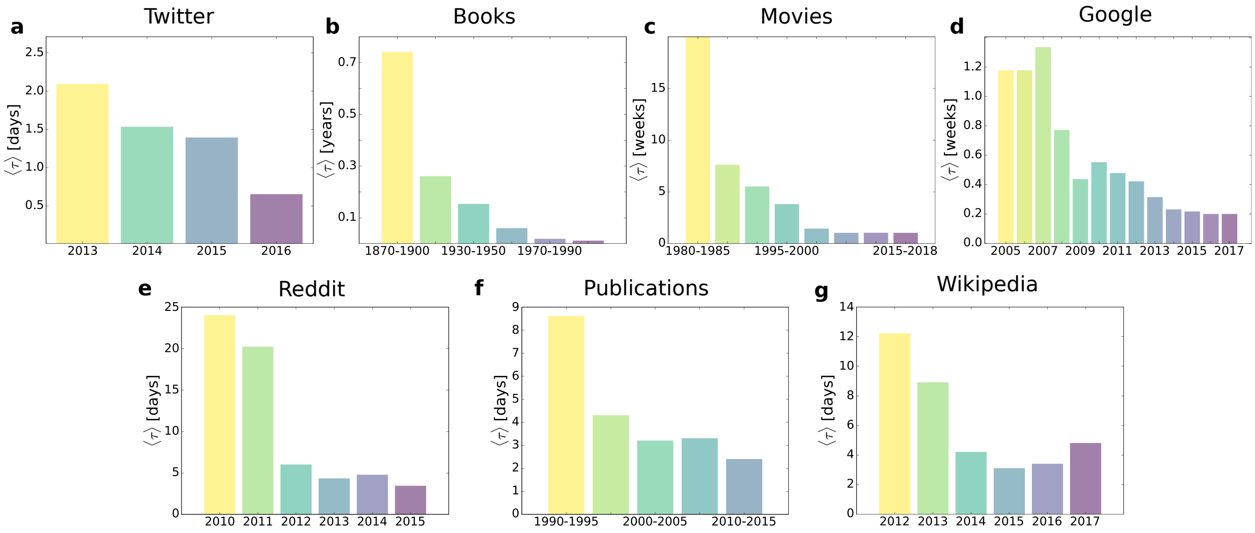

Supplementary Figure 7: **Average inter-event times:** The corresponding average times $\langle\tau\rangle$ between the events that are shown in 6 and Figure 1b. **a** Twitter, **b** Google Books, **c** Movies, **d** Google Trends, **e** Reddit, **f** Citations and **g** Wikipedia.

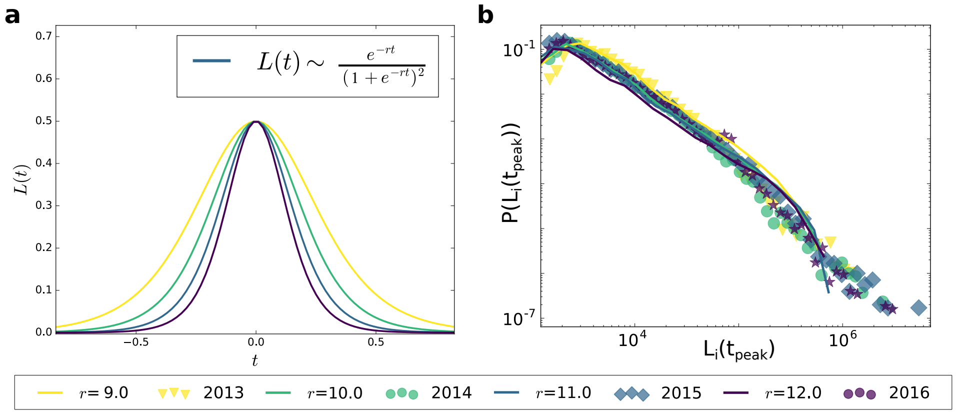

Supplementary Figure 8: **Details on the influence of r on the peak heights:** **a** The analytic expression obtained for a single topic under the variation of r, peak height stay stable while slopes increase. **b** The inset of Figure 3h magnified for a more accessible inspection.

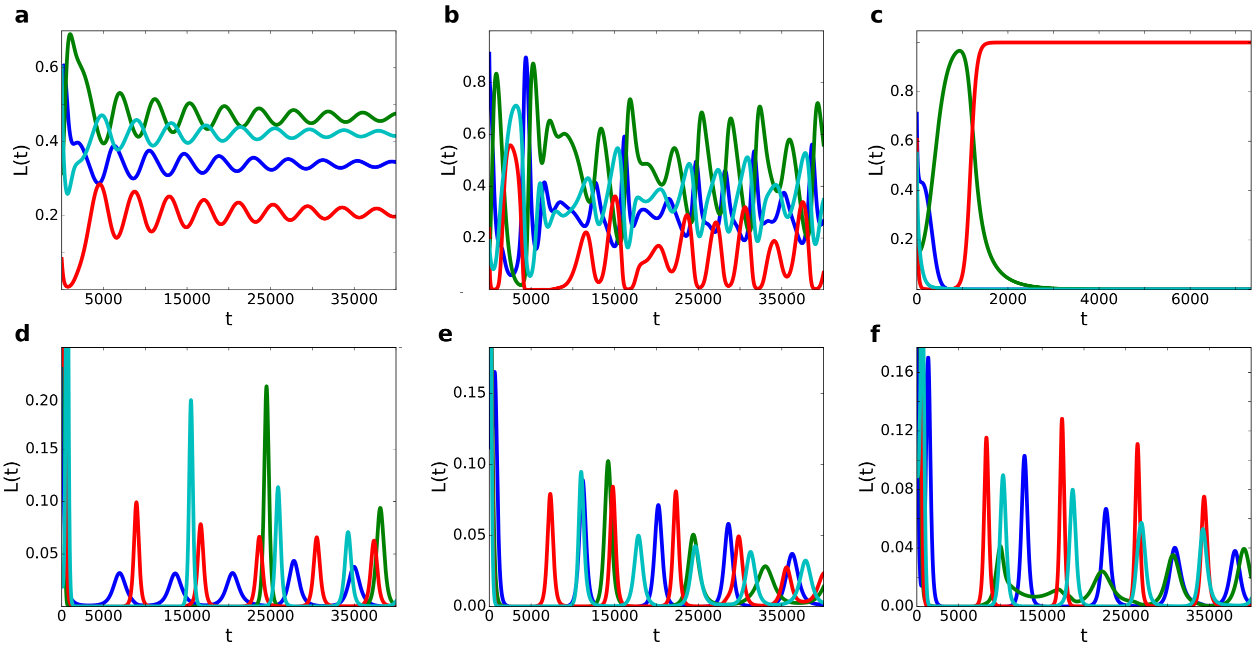

Supplementary Figure 9: **Scenarios of the Lotka-Volterra equations: a-c** Three phases of the classic Lotka-Volterra equations of $N = 4$ competing species (Eqs. (1)-(2)) **a** Coexistence of multiple species ($c = 0.3$), **b** chaotic dynamics between ($c = 1.0$) and **c** dominance of one species ($c = 3.0$). **d-f** The dynamics from the same parameters as above (Eq. (2)) but with the relations from our proposed model, including boringness effects (Eq. (3)). This model shows critical behavior for a broad range of parameter values.

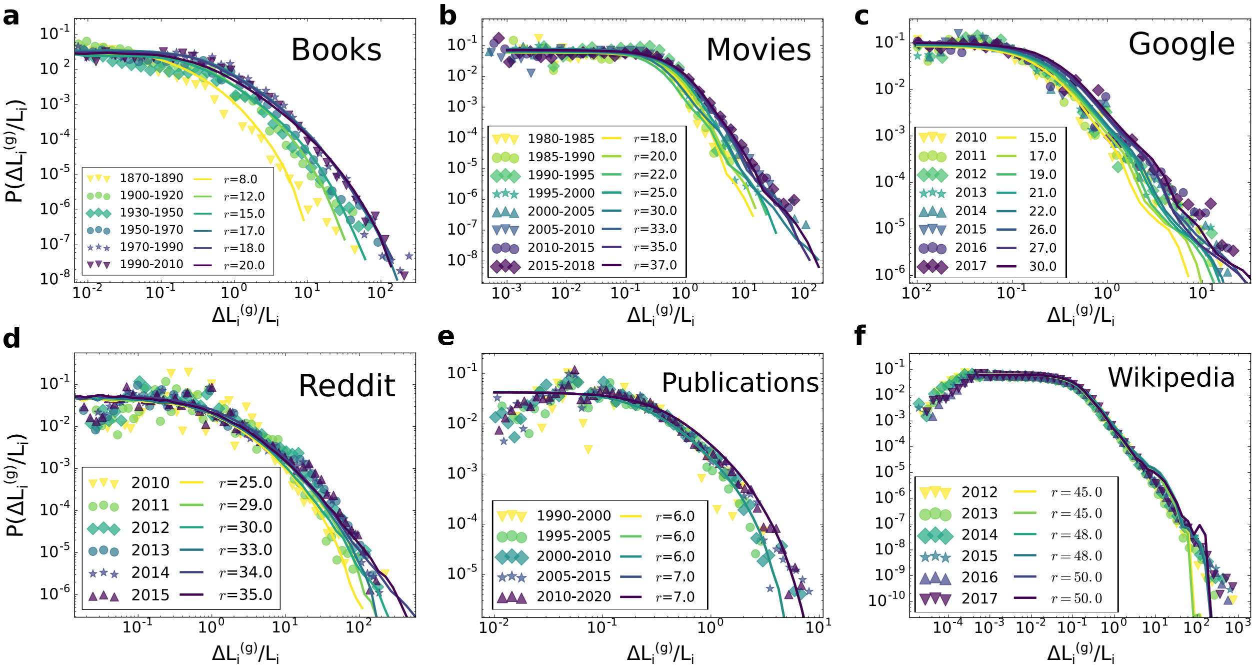

Supplementary Figure 10: **Simulation results for gain distributions:** **a** For the Google Books dataset we use $\alpha = 0.003$, $c = 2.4$, $N = 300$ and r as listed in the legend (KS-statistics, with more recent times: 0.16, 0.09, 0.11, 0.09, 0.07, 0.05). **b** For the Movie dataset we use $\alpha = 0.001$, $c = 2.4$, $N = 100$ and r as listed in the legend (KS-statistics: 0.12, 0.14, 0.20, 0.11, 0.10, 0.04, 0.04, 0.02). **c** For the Google Trends dataset we use $\alpha = 0.0005$, $c = 2.0$, $N = 100$ and r as listed in the legend (KS-statistics: 0.09, 0.10, 0.09, 0.10, 0.07, 0.09, 0.06, 0.07). **d** For the Reddit dataset we use $\alpha = 0.001$, $c = 5.4$, $N = 100$ and r as listed in the legend (KS-statistics: 0.09, 0.07, 0.11, 0.08, 0.07, 0.07). **e** For the citations dataset we use $\alpha = 0.003$, $c = 2.4$, $N = 300$ and r as listed in the legend (KS-statistics: 0.06, 0.05, 0.04, 0.10, 0.11). **f** For the Wikipedia dataset we use $\alpha = 0.0001$, $c = 2.4$, $N = 100$ and r as listed in the legend (KS-statistics: 0.18, 0.19, 0.18, 0.17, 0.16, 0.16).

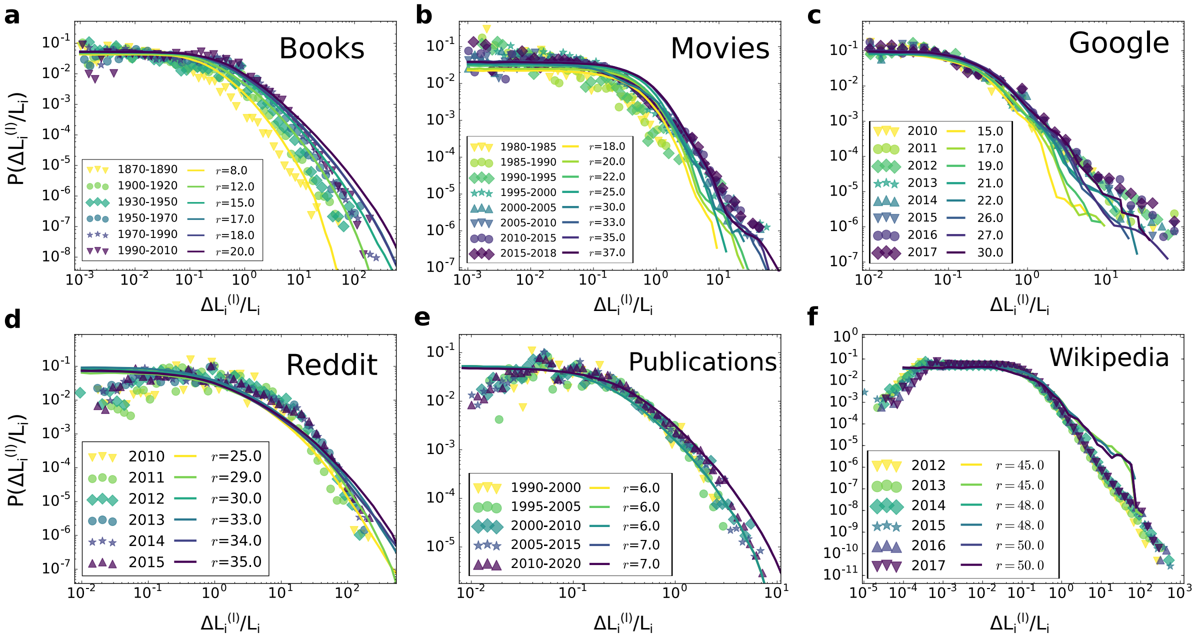

Supplementary Figure 11: **Simulation results for loss distributions: a-f** The results from the same simulations as shown in Figure 10 but for the loss-distributions in comparison to the empirical findings.

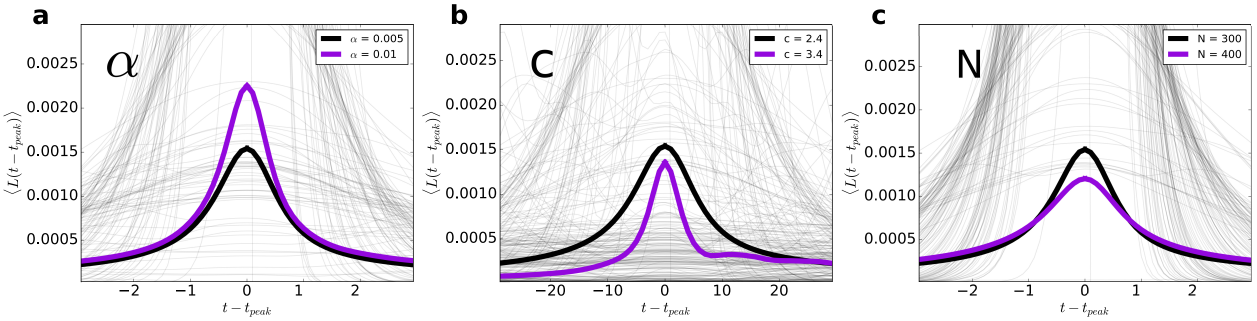

Supplementary Figure 12: **Variation of the other parameters:** The average trajectories of the original simulation ($\alpha = 0.005$, $c = 2.4$, $r = 12.0$ and N=300, in black) compared to the results under variation of one parameter at a time: **a** Shorter memory $\alpha = 0.01$ **b** Stronger competition $c = 3.4$ **c** Higher number of competitors $N = 400$. In none of the cases the observed developments of stable peak heights with increasing slopes can be reproduced.

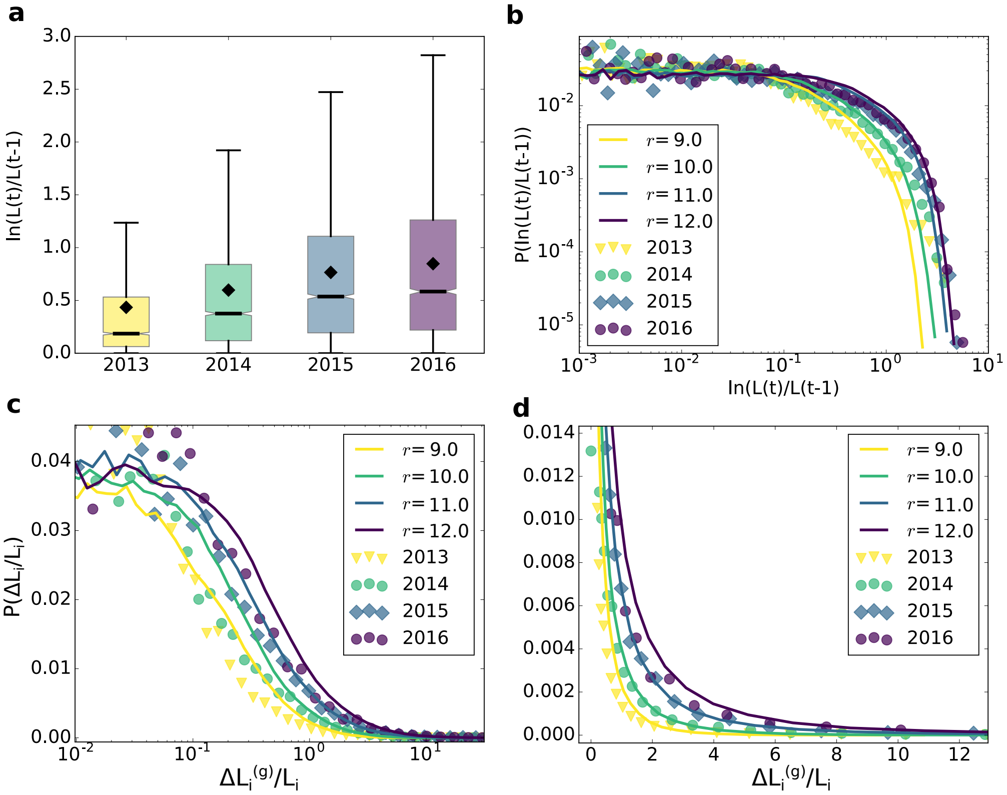

Supplementary Figure 13: **Alternative representations of the empirical and simulated data:** **a** The development of hashtag dynamics on twitter, quantified via the logarithmic change $\log(L(t)/L(t-1))$. **b** The same data as well as the results from the simulation as the logarithmic change $\log(L(t)/L(t-1))$. **c** The data shown in Figure 3 of the main text in a semi-log and **d** in a no-log representation.

Source	Timespan	Popularity proxy $L_{\mathrm{i}}(t)$	Res.	Origin
Twitter	2013-2016	hashtag occurrence	daily	`twitter.com`
Books	1870-2004	1- to 5-gram counts/book	yearly	`books.google.com/ngrams`
Movies	1980-2018	weekly gross per theater	weekly	`boxofficemojo.com`
Google	2010-2017	searches/max(searches)	weekly	`trends.google.com`
Reddit	2010-2015	comment counts per post	daily	`reddit.com`
Publications	1990-2015	citation counts per paper	monthly	`journals.aps.org`
Wikipedia	2012-2017	page views per article	daily	`dumps.wikimedia.org/ other/pagecounts-ez/`

Supplementary Table 1: **The datasets:** Table of data sources, observation time, the proxy used to measure popularity dynamics, and their origin.

Source	Sampling	Sample size
Twitter	Top 50 of each hour,<br>sorted by hourly volume	25031 (2013), 31012 (2014),<br>32945 (2015), 36703 (2016)
Books	Top 1000 of each year,<br>sorted by relative yearly volume	6900 (1870-1890), 9850 (1900-1920),<br>11120 (1930-1950), 11700 (1950-1970),<br>13100 (1970-1990), 12000 (1990-2004)
Movies	Popular movies of each week,<br>sorted by box-office sales	145 (1980-1985), 301 (1985-1990), 387 (1990-1995),<br>466 (1995-2000), 714 (2000-2005), 958 (2005-2010),<br>1012 (2010-2015), 688 (2015-2018)
Google	Top 20 of each month,<br>sorted by total queries	156 (2010), 201 (2011),<br>187 (2012), 240 (2013), 275 (2014),<br>285 (2015), 284 (2016), 295 (2017)
Reddit	Top 1000 of each month,<br>sorted by accumulated comments	6470 (2010), 7848 (2011), 9739 (2012),<br>10358 (2013), 10420 (2014), 10708 (2015)
Publications	More than 15 citations,<br>once in the observation window	482 (1990-1995), 906 (1995-2000), 1608 (2000-2005),<br>2154 (2005-2010), 2187 (2010-2015)
Wikipedia	Top 100 of every hour,<br>sorted by traffic per article	117623 (2012), 118375 (2013), 144970 (2014),<br>158752 (2015), 141032 (2016), 138031 (2017)

Supplementary Table 2: **Sample sizes from top-lists:** Sampling methods for popular items in the different datasets and the resulting sampling sizes N for the various observation windows.

Distribution	Twitter	Books	Movies	Google	Reddit	Publ.	Wikipedia
exponential	0.30	0.24	0.15	0.25	0.29	0.30	0.11
powerlaw	0.45	0.43	0.44	0.43	0.29	0.38	0.34
**lognormal**	0.02	0.04	0.02	0.04	0.05	0.03	0.03
Cauchy	0.27	0.23	0.20	0.23	0.28	0.22	0.20
normal	0.46	0.36	0.37	0.35	0.29	0.46	0.24
gamma	0.99	0.95	0.94	0.69	0.68	0.99	0.22
Pareto	0.06	0.07	0.05	0.05	0.07	0.18	0.08
logistic	0.33	0.28	0.25	0.29	0.29	0.34	0.22
uniform	0.98	0.95	0.94	0.89	0.77	0.98	0.76
Fisk	0.02	0.03	0.02	0.04	0.08	0.02	0.03
Weibull	0.05	0.09	0.07	0.15	0.10	0.08	0.08
truncated powerlaw	0.42	0.41	0.44	0.44	0.34	0.34	0.38

Supplementary Table 3: **Goodness of fit for various candidate distributions:** The distributions shown in Supplementary Figure 1 and the KS-statistics values for each dataset as a quantification of the quality of the fit. The log-normal has the lowest average value of 0.033 and by that represent the best suited distribution of this set of functions from descriptive statistics to fit our data. The Fisk, Weibull and Pareto distributions fit most of the data also very well.

	2013	2014	2015	2016
σ	1.96	1.97	2.03	2.11
μ	-1.91	-1.53	-1.02	-0.966
KS-statistics	0.018	0.027	0.024	0.015
p-value	0.22	0.01	0.03	0.43

Supplementary Table 4: **Fitted parameters for the Twitter dataset:** σ and μ are parameters of the log-normal distribution $P(x) = 1/(x\sigma\sqrt{2\pi})\exp\left[-(\ln x - \mu)^2/(2\sigma^2)\right]$. They are used as fitting parameters to minimize the KS-distance to the empirical distribution. The corresponding KS-statistics and p-values to each fit are listed below.

	1870-1890	1900-1920	1930-1950	1950-1970	1970-1990	1990-2010
σ	1.47	1.50	1.50	1.6	1.65	1.57
μ	-0.35	-0.17	0.084	0.17	0.38	0.18
KS-statistics	0.054	0.10	0.11	0.079	0.054	0.039
p-value	0.0	0.0	0.0	0.0	0.0	0.0

Supplementary Table 5: **Fitted parameters for the Google Books dataset:** σ and μ are parameters of the log-normal distribution $P(x) = 1/(x\sigma\sqrt{2\pi})\exp\left[-(\ln x - \mu)^2/(2\sigma^2)\right]$. They are used as fitting parameters to minimize the KS-distance to the empirical distribution. The corresponding KS-statistics and p-values to each fit are listed below.

	80-85	85-90	90-95	95-00	00-05	05-10	10-15	15-18
σ	0.92	0.78	0.81	1.01	1.03	1.15	1.20	1.19
μ	-0.01	-0.033	-0.035	-0.01	-0.007	-0.006	-0.005	-0.008
KS-statistics	0.04	0.04	0.04	0.04	0.03	0.02	0.03	0.02
p-value	0.3	0.2	0.04	0.007	0.002	0.05	0.004	0.03

Supplementary Table 6: **Fitted parameters for the Movie box-office dataset:** σ and μ are parameters of the log-normal distribution $P(x) = 1/(x\sigma\sqrt{2\pi})\exp\left[-(\ln x - \mu)^2/(2\sigma^2)\right]$. They are used as fitting parameters to minimize the KS-distance to the empirical distribution. The corresponding KS-statistics and p-values to each fit are listed below.

	2010	2011	2012	2013	2014	2015	2016	2017
σ	1.28	1.29	1.26	1.29	1.29	1.35	1.43	1.44
μ	-1.9	-1.8	-1.8	-1.8	-1.7	-1.7	-1.6	-1.6
KS-statistics	0.057	0.059	0.060	0.062	0.057	0.038	0.046	0.043
p-value	1.7e-6	1.4e-9	1.5e-9	1.9e-11	1.7e-10	1.0e-5	7.8e-7	3.7e-7

Supplementary Table 7: **Fitted parameters for the Google Trends dataset:** σ and μ are parameters of the log-normal distribution $P(x) = 1/(x\sigma\sqrt{2\pi})\exp\left[-(\ln x - \mu)^2/(2\sigma^2)\right]$. They are used as fitting parameters to minimize the KS-distance to the empirical distribution. The corresponding KS-statistics and p-values to each fit are listed below.

	2010	2011	2012	2013	2014	2015
σ	1.27	1.35	1.41	1.57	1.55	1.63
μ	1.36	1.57	1.91	2.01	2.00	1.90
KS-statistics	0.052	0.047	0.045	0.048	0.042	0.050
p-value	0.003	0.008	0.006	0.001	0.010	0.001

Supplementary Table 8: **Fitted parameters for the Reddit dataset:** σ and μ are parameters of the log-normal distribution $P(x) = 1/(x\sigma\sqrt{2\pi})\exp\left[-(\ln x - \mu)^2/(2\sigma^2)\right]$. They are used as fitting parameters to minimize the KS-distance to the empirical distribution. The corresponding KS-statistics and p-values to each fit are listed below.

	1990-1995	1995-2000	2005-2010	2010-2015	2015-2018
σ	0.98	1.01	1.10	1.32	1.58
μ	0.21	0.22	0.22	0.23	0.23
KS-statistics	0.056	0.036	0.032	0.038	0.029
p-value	0.04	0.01	0.001	0.00	0.0001

Supplementary Table 9: **Fitted parameters for the publications dataset:** σ and μ are parameters of the log-normal distribution $P(x) = 1/(x\sigma\sqrt{2\pi})\exp[-(\ln x - \mu)^2/(2\sigma^2)]$. They are used as fitting parameters to minimize the KS-distance to the empirical distribution. The corresponding KS-statistics and p-values to each fit are listed below.

	2012	2013	2014	2015	2016	2017
σ	1.42	1.42	1.45	1.43	1.42	1.41
μ	0.11	0.10	0.12	0.12	0.13	0.13
KS-statistics	0.027	0.026	0.029	0.028	0.027	0.025
p-value	0.0	0.0	0.0	0.0	0.0	0.0

Supplementary Table 10: **Fitted parameters for the Wikipedia dataset:** σ and μ are parameters of the log-normal distribution $P(x) = 1/(x\sigma\sqrt{2\pi})\exp\left[-(\ln x - \mu)^2/(2\sigma^2)\right]$. They are used as fitting parameters to minimize the KS-distance to the empirical distribution. The corresponding KS-statistics and p-values to each fit are listed below.

Distribution	Year	Parameters	KS-Statistics	p-value
$P(\Delta L_{\mathrm{i}}^{\text{(g)}}/L_{\mathrm{i}})$	**2016**	$\alpha = 0.005$, $c = 2.4$, $r = 12.0$	**0.01**	**0.85**
$P(\Delta L_{\mathrm{i}}^{\text{(l)}}/L_{\mathrm{i}})$	2016	$\alpha = 0.005$, $c = 2.4$, $r = 12.0$	0.07	0.00005
$P(\Delta L_{\mathrm{i}}^{\text{(g)}}/L_{\mathrm{i}})$	2015	$\alpha = 0.005$, $c = 2.4$, $r = 11.0$	0.03	0.003
$P(\Delta L_{\mathrm{i}}^{\text{(l)}}/L_{\mathrm{i}})$	2015	$\alpha = 0.005$, $c = 2.4$, $r = 11.0$	0.03	0.01
$P(\Delta L_{\mathrm{i}}^{\text{(g)}}/L_{\mathrm{i}})$	2014	$\alpha = 0.005$, $c = 2.4$, $r = 10.0$	0.05	0.0004
$P(\Delta L_{\mathrm{i}}^{\text{(l)}}/L_{\mathrm{i}})$	2014	$\alpha = 0.005$, $c = 2.4$, $r = 10.0$	0.08	0.0
$P(\Delta L_{\mathrm{i}}^{\text{(g)}}/L_{\mathrm{i}})$	2013	$\alpha = 0.005$, $c = 2.4$, $r = 9.0$	0.11	0.0
$P(\Delta L_{\mathrm{i}}^{\text{(l)}}/L_{\mathrm{i}})$	2013	$\alpha = 0.005$, $c = 2.4$, $r = 9.0$	0.12	0.0

Supplementary Table 11: **Goodness of the simulation:** Values from the Kolmorogov-Smirnov test for comparing two samples, one empirical from Twitter the other one from the simulation of the proposed model. The simulation meets the empirical distribution from Twitter very well even when only r is varied.

Supplementary note 1. The described developments of increasing relative gains and losses is not clearly pronounced for the datasets about scientific publications and Wikipedia (Figures 2 and Supplementary Figures 2-7). Only in the tails of the distributions is a small development towards higher values detectable (Supplementary Figures 3 and 4). In Supplementary Figures 6 and 7 an increase of extreme events is shown, but this corresponds only to the outer most events in the distributions, while the largest part stays very stable. There are two possible explanations for this: The systems change on even longer time scales than we investigated here and if we increased the window of data collection, we could see a more pronounced change. On the contrary, the more likely reason is that these systems follow mechanisms that are different from the other datasets in this work. We intentionally focus on areas which are pop-culture driven, where the increasing communication rates and especially the concept of boringness play a specifically big role. In these two systems knowledge is communicated, rather than news or entertainment being consumed. The bottleneck in these systems might not be the pure rate of information transfer and other mechanisms, than our simple model incorporates, govern their dynamics. In these systems other parameters could have changed such as the competition among scientist or their dynamics is mostly governed by external factors (22, 36). For the same reason the log-normal fit as well as our simulations do not match very well. Generally most systems are additionally exogenously driven and for a more realistic simulation one might have to combine endogenous and exogenous mechanisms, e.g. by adding an random external drive to the proposed model.
Nevertheless there are small hints to the same direction of acceleration as in the other datasets, but we are not capturing them fully, either by missing other important systemic mechanisms or by too narrow observation windows.
In the Wikipedia dataset we observe another difference to the other observations, the decreasing heights of maximal traffic on the articles in the inset of Figure 2g. Our interpretation of this is that the effects of proportional growth due to imitation is not the strongest driving force on Wikipedia, which makes the traffic less concentrated in the top group and a growing N causes a broadening of its allocation and the lowering of the maxima (as in Supplementary Figure 12c).

Supplementary note 2. To better understand the behavior of the model, we can show numerically how the boringness added to the existing Lotka-Volterra equations drives the system towards criticality. The competitive Lotka-Volterra equations

$$\frac{dL_{\mathrm{i}}(t)}{dt} = r_{\mathrm{i}} L_{\mathrm{i}}(t) \left(1 - \frac{L_{\mathrm{i}}}{K} - c \sum_{\mathrm{j}=1,\mathrm{j}\neq\mathrm{i}}^{N} a_{ij} L_{\mathrm{j}}(t) \right) , \tag{1}$$

can lead to chaotic behavior, if the following parameter set is used (31):

$$r_{\mathrm{i}} = \begin{pmatrix} 1.0 \\ 0.72 \\ 1.53 \\ 1.27 \end{pmatrix} , \quad a_{ij} = \begin{pmatrix} 1.0 & 1.09 & 1.52 & 0 \\ 0 & 1.0 & 0.44 & 1.36 \\ 2.33 & 0 & 1.0 & 0.47 \\ 1.21 & 0.51 & 0.35 & 1.0 \end{pmatrix} \tag{2}$$

and $K = 1.0$. Supplementary Figures 9a-c show the three distinct dynamical regimes. The global coupling parameter c in Eq. (11) is increased from 0.3 to 3.0. Besides coexistence and dominance for small and large values of c respectively, for $c = 1.0$ the system is at the critical point and shows chaotic dynamics (31). Adding the boringness term yields

$$\frac{dL_{\mathrm{i}}(t)}{dt} = r_{\mathrm{i}} L_{\mathrm{i}}(t) \left(1 - \frac{r_c}{K} \int_0^t e^{-\alpha(t-t')} L_{\mathrm{i}}(t') dt' - c \sum_{\mathrm{j}=1,\mathrm{j}\neq\mathrm{i}}^{N} a_{ij} L_{\mathrm{j}}(t) \right), \tag{3}$$

with $K = r_c = 1.0$. Then, the critical behavior can be observed in all three parameter regimes (Figures S9d-f). The two states, coexistence and dominance of a single topic are constantly driven towards each other, where imitation prevents coexistence and boringness does not allow the dominance of a single topic. This can possibly explain the broad distributions resulting in systems that undergo self-organized criticality and is subject of future research.

Supplementary note 3. The simplistic nature of the model makes the eigenvalue of the Jacobian matrix of Eqs. (1)-(2) with just two competing topics ($N = 2$, $r_c = 1$ and $K = 1$) analytically tractable. For the class of systems that a setup of just two competing topics falls into, it has been shown that they can undergo a Hopf bifurcation towards self-sustained oscillations (32). The minimal system

$$\frac{dL_1(t)}{dt} = rL_1(t)\left(1 - Y_1(t) - cL_2(t)\right) \tag{4}$$

$$\frac{dY_1(t)}{dt} = L_1(t) - \alpha Y_1(t) \tag{5}$$

$$\frac{dL_2(t)}{dt} = rL_2(t)\left(1 - Y_2(t) - cL_1(t)\right) \tag{6}$$

$$\frac{dY_2(t)}{dt} = L_2(t) - \alpha Y_2(t) \tag{7}$$

has a fixed point ($L_1^* = \frac{\alpha}{1+\alpha c} = L_2^*$), where we can evaluate the Jacobian matrix and compute the relevant eigenvalue

$$\lambda = \frac{1}{2(1+\alpha c)} \left(-\alpha - \alpha^2 c + \alpha c r + \sqrt{(-\alpha - \alpha^2 c + \alpha c r)^2 + 4(-\alpha r + \alpha^3 c^2 r)} \right). \tag{8}$$

This can be further approximated by dropping quadratic terms of α (which is chosen to be small)

$$\lambda \approx \frac{1}{2(1+\alpha c)} \left(-\alpha + \alpha c r + 2i\sqrt{\alpha r} \right). \tag{9}$$

Near the fixed point its imaginary part gives an estimate for the relation of the rate r to the frequency of oscillating topics by

$$\mathrm{Im}(\lambda) \sim \frac{\sqrt{\alpha r}}{1+\alpha c}. \tag{10}$$

This relationship shows the positive proportionality of the frequency to the rate r, which we can also observe in the simulation of the full system.
